# Role of the Microbiome in Regulating Bone Metabolism and Susceptibility to Osteoporosis

**DOI:** 10.1007/s00223-021-00924-2

**Published:** 2021-12-06

**Authors:** Owen Cronin, Susan A. Lanham-New, Bernard M. Corfe, Celia L. Gregson, Andrea L. Darling, Kourosh R. Ahmadi, Philippa S. Gibson, Jon H. Tobias, Kate A. Ward, Maria H. Traka, Megan Rossi, Claire Williams, Nicholas C. Harvey, Cyrus Cooper, Kevin Whelan, André G. Uitterlinden, Paul W. O’Toole, Claes Ohlsson, Juliet E. Compston, Stuart H. Ralston

**Affiliations:** 1 Rheumatic Diseases Unit, Western General Hospital, Crewe Road South, Edinburgh, EH4 2XU UK; 2 Centre for Genomic and Experimental Medicine, Institute of Genetics and Cancer, University of Edinburgh, Crewe Road South, Edinburgh, EH4 2XU UK; 3 Nutrition, Food and Exercise Sciences Department, School of Biosciences and Medicine, Faculty of Health and Medical Sciences, University of Surrey, Guildford, GU2 7XH UK; 4 Population Health Sciences Institute, Human Nutrition Research Centre, Faculty of Medical Sciences, Newcastle University, Newcastle, NE2 4HH UK; 5 MRC Integrative Epidemiology Unit, Bristol Medical School, University of Bristol, Bristol, UK; 6 Musculoskeletal Research Unit, Translational Health Sciences, Bristol Medical School, University of Bristol, Bristol, UK; 7 Department of Nutritional Sciences, King’s College London, London, UK; 8 MRC Lifecourse Epidemiology Centre, University of Southampton, Southampton, UK; 9 NIHR Southampton Biomedical Research Centre, University of Southampton and University Hospital Southampton NHS Foundation Trust, Southampton, UK; 10 Food Databanks National Capability, Quadram Institute Bioscience, Norwich Research Park, Norwich, NR4 7UQ UK; 11 Molecular Gastroenterology Research Group, Academic Unit of Surgical Oncology, Department of Oncology and Metabolism, University of Sheffield, Beech Hill Road, Sheffield, S10 2RX UK; 12 NIHR Oxford Biomedical Research Centre, University of Oxford, Oxford, UK; 13 Department of Internal Medicine, Erasmus Medical Centre, Rotterdam, The Netherlands; 14 School of Microbiology and APC Microbiome Ireland, University College Cork, Room 447, Food Science Building, Cork, T12 K8AF Ireland; 15 Sahlgrenska Osteoporosis Centre, Center for Bone and Arthritis Research, Institute of Medicine, Sahlgrenska Academy, University of Gothenburg, Gothenburg, Sweden; 16 Department of Medicine, Cambridge Biomedical Campus, Cambridge, UK

**Keywords:** Osteoporosis, Microbiome, Immunology, Probiotics

## Abstract

The human microbiota functions at the interface between diet, medication-use, lifestyle, host immune development and health. It is therefore closely aligned with many of the recognised modifiable factors that influence bone mass accrual in the young, and bone maintenance and skeletal decline in older populations. While understanding of the relationship between micro-organisms and bone health is still in its infancy, two decades of broader microbiome research and discovery supports a role of the human gut microbiome in the regulation of bone metabolism and pathogenesis of osteoporosis as well as its prevention and treatment. Pre-clinical research has demonstrated biological interactions between the microbiome and bone metabolism. Furthermore, observational studies and randomized clinical trials have indicated that therapeutic manipulation of the microbiota by oral administration of probiotics may influence bone turnover and prevent bone loss in humans. In this paper, we summarize the content, discussion and conclusions of a workshop held by the Osteoporosis and Bone Research Academy of the Royal Osteoporosis Society in October, 2020. We provide a detailed review of the literature examining the relationship between the microbiota and bone health in animal models and in humans, as well as formulating the agenda for key research priorities required to advance this field. We also underscore the potential pitfalls in this research field that should be avoided and provide methodological recommendations to facilitate bridging the gap from promising concept to a potential cause and intervention target for osteoporosis.

## Introduction

This viewpoint article summarizes current knowledge in the microbiome field and identifies research priorities to advance microbiome research in osteoporosis and bone health. Most of the studies in this field have focussed on analysis of the microbiome content of faeces or stool samples which are considered to represent the gut microbiome. Therefore, within this article we will use the broad term “gut microbiome” to describe this research while acknowledging that there is actually very little information on the microbiome content in different parts of the intestine. The overall goal is to improve our current understanding of the relationship between the microbiome and bone health, and to translate this knowledge into actionable recommendations to prevent the development of osteoporosis or to find a cure for patients with this condition.

### The Gut Microbiome and Osteoporosis: An Appetite for Discovery and Translation

It has been estimated that nine million fragility fractures occur worldwide each year, which are associated with significant morbidity and mortality [1–3]. The therapeutic options for osteoporosis that reduce fracture risk have expanded as anabolic agents have become available [4, 5]. Despite this, primary and secondary fracture prevention remains sub-optimal [6]. The reasons for this are multi-factorial but include fear of adverse effects of medication and poor co-ordination of healthcare systems [7–9]. A frequent question asked by patients is; “are there any supplements, changes in diet or lifestyle change that I can make instead of taking drugs?” [10].

**Fig. 1:**
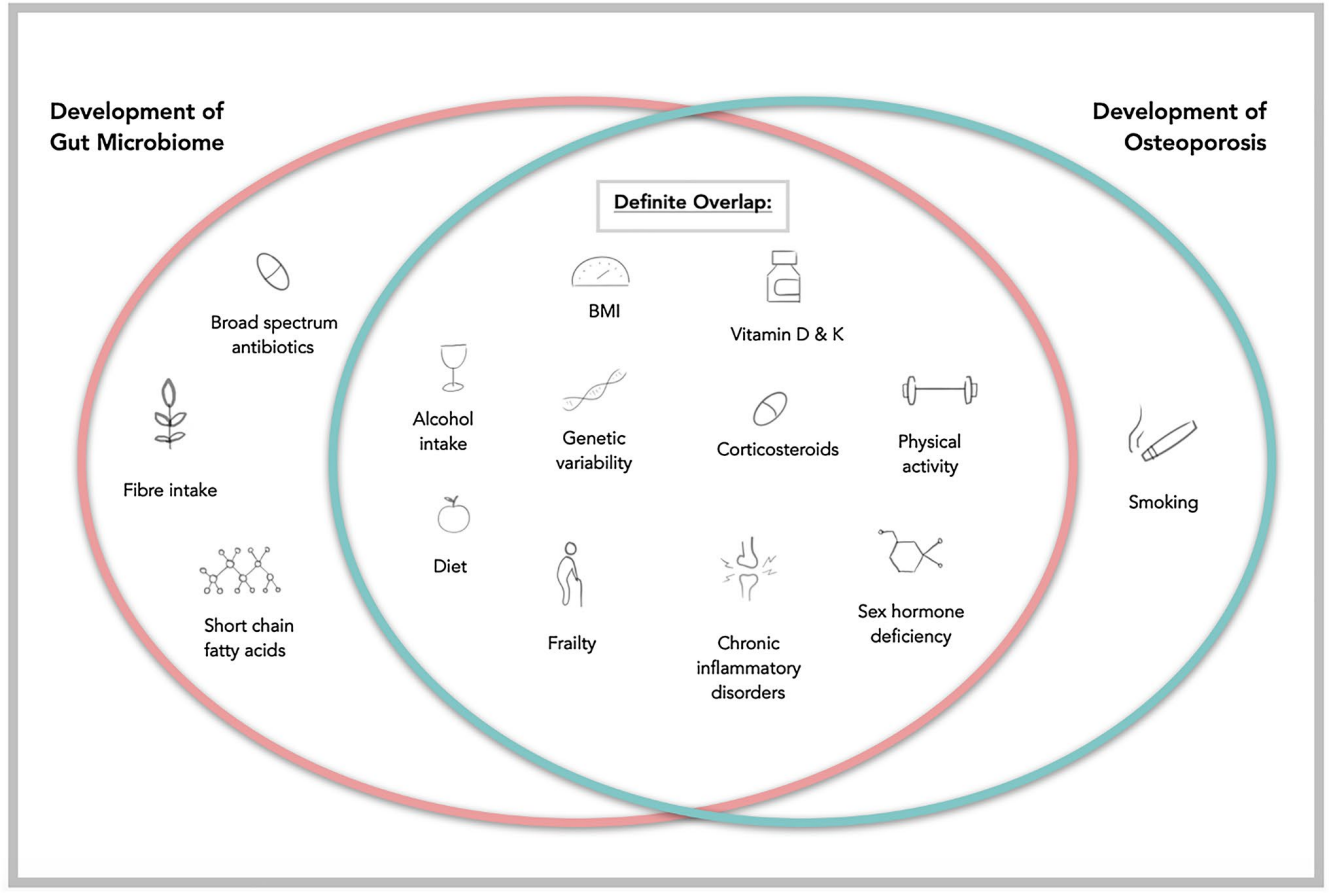
Contributing factors that influence the development of the gut microbiota and the pathogenesis of osteoporosis. While many factors influencing the microbiome and development of osteoporosis overlap, the effects of others are unknown or are under active investigation

Over the last two decades, the development and availability of high-throughput next-generation genome sequencing technologies has facilitated the characterization of the array of micro-organisms that reside within the human intestine, known as the gut microbiota. Our understanding of the collective genome of these microbes, known as the gut microbiome, is ever-expanding. It is now well-established that the composition, and in many cases, the functional potential and metabolic activity of the gut microbiome is heavily influenced by factors such as diet [11], medication use with drugs such as glucocorticoids and broad-spectrum antibiotics [12, 13], as well as frailty and ageing [14]. Co-incidentally, many of these variables also contribute to the pathogenesis of osteoporosis (Fig. 1) [15]. Whilst well-established mechanisms exist to explain how these factors affect fracture risk, it is pertinent to explore the influence that the gut microbiome may have both directly on bone architecture and metabolism, and indirectly through its interactions with ‘traditional’ risk factors for osteoporosis.

Historically, the relationship between the gut microbiota and bone health has received scant attention in the literature. However, in less than five years, mechanistic studies in animals have progressed to association studies in humans; and onwards to clinical trials of the effects of probiotics in women with reduced bone mineral density (BMD) [16–19]. The acceleration in microbiome research in bone metabolism is not unique since interest has increased exponentially across many other medical specialties. Exploring the influence of the microbiota on bone health provides a novel and exciting means of advancing our understanding of the pathogenesis of osteoporosis and potentially unearthing new preventative and therapeutic targets.

## Knowledge to Date: Animal Studies

A new term of osteo-microbiology was introduced for the rapidly emerging research field of the role of the gut microbiota in bone health [20]. Much of the interest stemmed from initial findings in murine studies [21]. The available literature may be broadly compartmentalized across three categories of animal studies; Germ-free (GF) murine studies; antibiotic/probiotic/prebiotic intervention studies; and models of postmenopausal osteoporosis.

### Germ-Free (GF) Murine Models

One of the earliest studies, by Sjogren et al. demonstrated that mice raised in GF environments, who therefore lack a gut microbiota, exhibited higher trabecular bone mass and reduced osteoclastic activity compared to conventionally raised mice [22]. Furthermore, repopulation of the gut of the GF mice with microbiota from the distal intestine of conventionally-raised mice led to a normalization in bone mass. This suggests that the presence of the gut microbiota is required for normal osteoclastic activity. Further studies by this group demonstrate that the ability of the gut microbiota to increase osteoclastogenic RANKL and TNF-α activity in bone is dependent on Nod-like receptors NOD1 and NOD2 in the host [23]. NOD-like receptors are pattern recognition receptors, representing a critical component of innate immunity, supporting the hypothesis that the gut microbiota may affect bone turnover in an immune-mediated manner.

Whilst an increase in bone mass in GF mice has been replicated by others [24], the degree of reduction or normalization of bone mass in GF mice following reconstitution of the intestine with micro-organisms has not been consistent across all investigations. A study by Quach et al. did not identify significant change in trabecular bone volume in GF mice reconstituted with faeces from human vegetarians and omnivores, nor with caecal microbiota from conventionally raised mice [25]. The authors of this study highlight the genetic make-up of the mouse model and selection of donor microbial communities as key considerations that may influence the outcomes on bone health following microbial colonization of GF animals.

GF mouse models have also been used to investigate the mediators of microbiota-dependent bone mass regulation. Two separate studies in GF mice have implicated the production of short chain fatty acids (SCFA) such as butyrate and acetate by the gut microbiota as an important mediator of bone formation [26, 27]. Based on their work in GF *CB6F1* mice, Yan and colleagues propose that anabolic effects on bone following chronic colonization of GF mice (8 months) with a pathogen-free microbiota are possibly mediated by insulin-like growth factor 1 (IGF-1) production [27]. Additionally, antibiotic treatment of conventionally raised mice led to a reduction in circulating IGF-1 and concurrent inhibition of bone formation. Provision of SCFA to mice who received antibiotics restored IGF-1 and bone mass to levels present in mice that were not treated with antibiotics. The potential of microbial-derived SCFA, in particular butyrate, to mediate bone formation was further supported in another GF and antibiotic-treated mouse study [26]. Here, the anabolic effects of parathyroid hormone (PTH) were dependent on an intact microbiota. Strikingly, nutritional supplementation of butyrate in antibiotic-treated mice led to restoration of the ability of daily PTH injections to stimulate bone formation and increase trabecular thickness.

### Antibiotic-Intervention Studies

Antibiotics are an established effector of the human gut microbiome and when not used judiciously, can have deleterious effects on host health, including life-threatening infection with *Clostridioides difficile*.[28, 29] The long-term sequelae of early-life consumption of antibiotics is the subject of much interest. In the preterm infant, antibiotic use can lead to reduced intestinal species diversity and richness, in addition to long-lasting carriage of multi-drug resistant organisms [30]. Association studies indicate that antibiotic use in childhood increases the risk of developing atopy and autoimmune inflammatory disorders such as juvenile idiopathic arthritis and rheumatoid arthritis in later life [31–33].

Studies in rodents have revealed that repeated depletion of the gut microbiota in early life by pulsed oral intake of broad-spectrum antibiotics can lead to increased bone mineral content and BMD [34]. The authors suggest that the increased BMD observed in mice receiving the widely used, broad-spectrum beta-lactam, amoxicillin, may be due to a greater abundance of oxalate-degradation genes present in the gut microbiome post-antibiotic treatment. A reduction in levels of oxalate, a major calcium-binding anion in the intestinal lumen, could facilitate a greater availability and absorption of calcium from the intestines, enhancing bone mineralisation. This theory was supported by the presence of lower levels of oxalate in the faecal matter of mice receiving amoxicillin.

Another potential mechanism by which alteration of the gut microbiome may affect bone health relates to the microbial synthesis and luminal availability of vitamin K_2_ (menaquinone). Warfarin, the widely used vitamin K antagonist, has long been associated with a small increase in the risk of osteoporotic fractures in adults [35, 36]. While vitamin K can affect bone quality through several mechanisms, the presence of vitamin K-dependent γ-carboxyglutamic acid residues is crucial for osteocalcin production and bone formation [37]. Using a metagenomic-based sequencing approach, Guss and colleagues demonstrated that disruption of the gut microbiota of *C57BL/6 J* inbred mice by broad spectrum antibiotics (ampicillin and neomycin) led to a reduced abundance of vitamin K-producing genes within the gut microbiome (*MenB, MenC, MenG*). This resulted in a subsequent deterioration in bone mineral crystallinity.[38] Their work was supported by the finding of reduced availability of menaquinone within the intestine, liver and kidney of the antibiotic-treated mice. This study provides an excellent example of how metagenomic sequencing, combined with metabolite quantification in biological samples, can provide valuable insight into functional metabolic capacity and activity, allowing us to understand how the gut microbiome can affect processes relevant to bone health.

### Models of Postmenopausal Osteoporosis

To investigate whether the gut microbiome plays a role in the pathogenesis of postmenopausal osteoporosis, GF mice studies have frequently been used in combination with ovariectomy or with oestrogen deficiency induced by gonadotrophin-releasing hormone analogues (GnRH).

One of the more striking examples of this is provided by Li et al. [24] This study demonstrated that upregulation of osteoclastogenic cytokines in the bone marrow (IL-17, TNF-α, and RANKL) and trabecular bone loss did not occur in GF mice in response to oestrogen deficiency but did occur in conventionally raised mice [24]. This was supported by two other studies in ovariectomized rats, where perturbation of the indigenous microbiota with doxycycline attenuated sex steroid deficiency-induced changes in bone mineral content and mechanical properties [39, 40]. Furthermore, in the study of Li and colleagues, twice-weekly probiotic supplementation of the indigenous microbiota of sex-steroid deficient mice with *Lactobacillus rhamnosus* provided protection against bone loss, whereas supplementation with a non-probiotic strain of *Escherichia coli* did not.

Lawenius and colleagues have examined the effects of a novel probiotic, pasteurized *Akkermansia muciniphila,* on ovariectomy-induced bone loss [41]. This mucin-degrading micro-organism provides an attractive option as a next-generation probiotic [42]. It is known to improve barrier function in the gut, has led to improved metabolic status in mice and its abundance is inversely associated with obesity and associated with the degree of athletic fitness in humans [43–45]. In the ovariectomized mouse model, supplementation with *A. muciniphila* for four weeks led to reduced fat mass accumulation, mirroring previous studies. However, in comparison to mice who received sham surgery, probiotic treatment did not confer any protective benefit on bone health. Specifically, *A. muciniphila* reduced bone mass in gonadal-intact mice who underwent sham ovariectomy but did not attenuate bone loss in ovariectomized mice.

In summary, the data from animal studies provide evidence to support a role of the gut microbiome on the regulation of bone mass attainment and bone remodelling. The evidence also suggests that some of the effects of oestrogen deficiency on bone health are microbiota-dependent. The effects of microbiota alteration or depletion by antibiotics is less consistent and requires further study. However, caution is needed when attributing direct causation to the effects of specific microbes or microbial communities to bone formation or resorption. This is particularly true of GF animal studies, since these conditions cannot be replicated in humans and are not directly translatable [46].

## Knowledge to Date: Human Studies

### Human Observational Studies

Several observational studies examining the relationship between BMD and the gut microbiota have recently been published [17, 47, 48]. All of these have used 16 s ribosomal RNA amplicon sequencing to analyse the gut microbiota. The largest study, published by Das et al., [17] examined variation in the gut microbiota according to BMD measurements in 181 adults; 60 with normal BMD, 61 with osteopenia and 60 with osteoporosis. The mean age of participants was 64 years and the cohort was predominantly female. Microbial diversity in the gut had previously has been show to correlate with frailty in elderly residents of residential homes [14], but did not associate with BMD in this younger cohort of community dwellers. A large number of potential confounders were recorded and after controlling for these, five taxa remained significantly different between the normal BMD and osteopenic/osteoporotic groups.

Interestingly, a study of the EPIC cohort (European Prospective Investigation into Cancer and Nutrition) has shown an association between vegan and vegetarian diets and fracture incidence [49]. Although the researchers did not specifically interrogate the effects of these diets on the gut microbiota, this is clearly an area of research interest given the increasing popularity of vegan and plant-based diets in young, female individuals. Furthermore, in the EPIC-Norfolk cohort, observational analysis of the relationship between the Mediterranean diet and fracture incidence demonstrated that greater adherence to the diet was associated with a significant reduction in fracture risk [50]. Although, it is not possible to attribute causation to this, long-term adherence to the Mediterranean diet is known to directly impact the gut microbiota with resultant health benefits for the host [11, 51].

### Interventional Studies

There have been several randomized controlled trials in humans investigating the effects of probiotic supplementation on bone health.

Nissen and colleagues performed a double-blind, placebo-controlled trial examining the effect of the probiotic *Lactobacillus reuteri* ATCC PTA 6475 on bone loss of women with reduced bone mass (mean age 76 years) [18]. This trial built upon convincing evidence from ovariectomized mice, where supplementation with *L. reuteri* ATCC PTA 6475 protected against bone resorption and loss associated with oestrogen deficiency [52]. Seventy women completed the study which involved taking the probiotic at a dose of 1 × 10^10^ colony-forming units (CFU) per day or placebo. After one year, those receiving the probiotic supplement experienced a reduced loss of volumetric BMD at the tibia compared to those taking placebo (mean relative change − 0.83% [95% CI − 1.47 to − 0.19%], vs. − 1.85% [95% CI − 2.64% to − 1.07%]; intention to treat analysis). Subsequently, the same group of authors performed a metabolomic-based analysis of serum samples from participants in both treatment groups using liquid chromatography—tandem mass spectrometry to identify possible mechanisms for these effects. The authors identified 97 metabolites involved in multiple processes, including amino acid, peptide, and lipid metabolism which showed trends for differences between the treatment groups, but none remained significant after correction for multiple testing [53]. Accordingly the mechanisms by which *Lactobacillus reuteri* affects bone mass remains incompletely understood at present.

The effects of *Lactobacilli* probiotics for improvement of bone health was further studied using a combination of three *Lactobacillus* strains (*L*. *paracasei* DSM 13434, *L. plantarum* DSM 15312, and *L. plantarum* DSM 15313) in a multicentre, randomized, double-blind, placebo-controlled trial in Sweden [19]. This trial built on evidence in ovariectomized mice, where supplementation with the same mix of three *Lactobacillus* strains protected against bone loss associated with oestrogen deficiency [54]. Early postmenopausal women were randomized on a 1:1 basis to combination *Lactobacillus* probiotic at a daily dose of 1 × 10^10^ CFU or placebo for 12 months. 234 randomized participants completed the study with the *Lactobacillus* probiotic recipients experiencing minimal loss in lumbar spine BMD over 12 months (mean percentage change − 0.01% [95% CI − 0.50 to 0.48]), whereas those receiving placebo experienced a larger reduction in BMD (− 0.72% [95% CI − 1.22 to − 0.22%]).

In a further randomised trial performed by Takimoto and colleagues [55], 76 healthy postmenopausal Japanese women were allocated to receive the probiotic *Bacillus subtilis* (C-3102) or a matching placebo for a period of 24 weeks. There was a significant increase in total hip BMD of about 1.5% in the C-3102 group compared with the placebo group, but no significant difference in lumbar spine BMD. There was a trend for lower levels of the bone resorption markers urine NTX and serum TRAP 5b in the probiotic group at 12 weeks but this was not sustained at 24 weeks. The authors also noted changes in the relative abundance of various gut microbiota species in the C-3102 group compared with placebo. Consistent decreases in *Fusobacteria* species were noted at 12 and 24 weeks whereas the abundance of *Bifidobacterium* increased at 12 weeks in the C-3102 group. This led the authors to speculate that the decrease in *Fusobacteria* and increase in *Bifidobacterium* might favourably influence bone density by decreasing production of cytokines which regulate bone resorption. Nonetheless the authors also pointed out that overall, there was no significant correlation between the relative abundance of individual bacterial species and BMD or bone turnover markers in the study population.

In another study, Lambert and colleagues [56] investigated the effects of isoflavones and probiotics on bone health in a randomised placebo-controlled trial involving 85 postmenopausal women with osteopenia on DEXA with an average age of just over 60 years. The isoflavones and probiotics were delivered in the form of red clover extract (RCE) given twice daily for 12 months and the effects of this was compared with an identical placebo. The probiotic component of the active intervention comprised a heterogeneous culture of lactic acid producing bacteria. Participants in both groups were given calcium (1200 mg daily), magnesium (550 mg daily) and calcitriol (25 mcg daily) throughout the study. The RCE was found to significantly reduce bone loss at the lumbar spine, femoral neck and trochanter compared with placebo. In the RCE group, the bone resorption marker serum CTX-I was slightly but significantly reduced at 12 months whereas no significant change was detected in the placebo group. No significant change was detected in the bone formation marker P1NP during the study. Serum osteoprotegerin concentrations increased during the study to a similar extent in both groups. The authors concluded that the intervention was effective in slowing postmenopausal bone loss over a 12 month period but the study design did not allow them to determine whether it was the probiotic, the isoflavones or the combination of both interventions that was responsible for the effect observed.

Jafarnejad and colleagues [57] conducted a randomised placebo controlled trial of probiotics in 50 postmenopausal Iranian women with osteopenia for a 6-month period. The probiotic intervention consisted of a capsule containing seven different strains of probiotics (four species of *Lactobacillus*, two species of *Bifidobacterium* and one of *Streptococcus thermophilus*). The authors measured changes in spine and hip BMD as well as a large number of biochemical markers of bone metabolism at baseline and at 6 months. No significant changes in BMD were observed in either group. There were nominally significant falls from baseline in serum TNFα, bone specific ALP, CTX and PTH in the probiotic group compared with placebo groups from baseline, analysed by ANCOVA, but no correction for multiple testing was applied. The authors concluded that probiotics may have reduced bone turnover and production of the pro-inflammatory cytokine TNFα which also acknowledging that the sample size was small and the duration of the study relatively brief.

In summary, the available evidence suggests that probiotic supplements can attenuate bone loss in postmenopausal women, although the studies investigating this have been short term and individually have had small sample sizes. Moving forward, it will be important to conduct larger scale studies to evaluate if the skeletal response differs with different types of probiotic and also to determine of the effects are sustained in the longer term. Few human studies have examined the effect of dietary manipulation of the gut microbiota on bone health. However, candidate nutrients such as fibre and isoflavones have been identified as potential effectors [56, 58].

### Vitamin D and the Microbiome

Vitamin D receptors are highly expressed within the gastrointestinal tract, particularly in the small intestine and colon [59]. However, expression may be reduced in disorders associated with reduced gut microbiota diversity such as inflammatory bowel disease [60]. In a recent cross-sectional analysis of 567 older men, serum levels of calcitriol, the active hormone of vitamin D accounted for between 2 and 5% of the variance in the microbial diversity in the gut [61]. This investigation identified eight specific taxa, many from the phylum *Firmicutes,* that positively correlated with levels of calcitriol. Furthermore, participants with higher levels of circulating calcitriol were more likely to have a microbiota associated with SCFA production (butyrate), suggesting that the gut microbiota may influence vitamin D bioavailability.

It has been postulated that probiotics exert a role in increasing circulating 25-hydroxyvitamin D. A randomized controlled trial of 9-weeks of supplementation with *Lactobacillus reuteri* (NCIMB 30,242) in healthy participants led to a 25% increase in serum 25-hydroxyvitamin D in those receiving the supplement [62]. Conversely, a small, randomized controlled trial examining dose response of oral vitamin D supplementation in 20 adults with vitamin D deficiency demonstrated differential, dose-dependent increases in the relative abundance of *Bacteroides* after 8 weeks of supplementation [63].

The existing evidence suggests that the gut microbiome and probiotics may alter vitamin D metabolism within the gut, but research also indicates that increased oral vitamin D intake may affect the composition of the gut microbiota. Both these possibilities require further investigation given the widespread nature of vitamin D insufficiency in all age groups of the UK population.[64]

## Future Directions

**Fig. 2:**
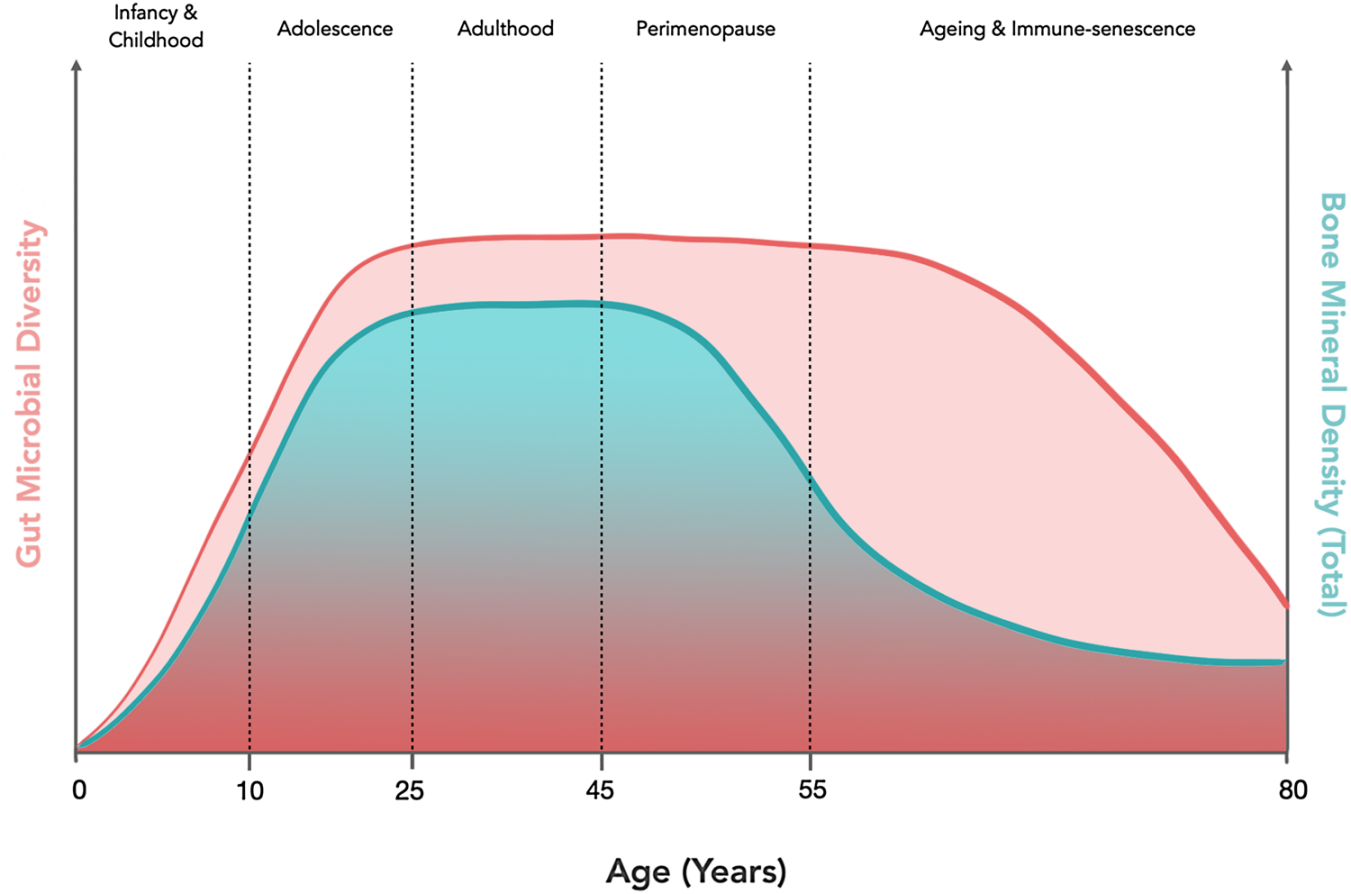
Trajectory of bone mineral density and gut microbiota diversity across the life course. The trajectory of bone mass throughout life is similar in many respects to changes in diversity of the gut microbiota throughout the lifespan with the greatest changes occurring during infancy and adolescence

During the Royal Osteoporosis Society workshop, invited experts in the areas of osteoporosis, nutrition and microbiome research discussed the key knowledge gaps in the area of microbiome research as it applies to bone metabolism and osteoporosis. Specifically, the group considered the populations of interest, the key clinical questions to be addressed and the study designs that might be employed to advance the field (Table 1). A need for prospective, longitudinal observational studies was identified since both bone health and the gut microbiome are subject to temporal changes across the life course and the relationship between them at critical junctures of development and physiological change needs to be investigated (puberty, illness, menopause, frailty; see Fig. 2).

**Table 1:** A list of potential research questions to understand the impact of the gut microbiota on bone health and osteoporosis

	Question of interest	Possible mechanism of investigation
1	Are nutrition and lifestyle effects on the gut microbiota in early life (birth to adolescence) related to peak bone mass (PBM) attainment?	Longitudinal cohort study
2	Are nutritional and lifestyle effects on the gut microbiota in early life (birth to adolescence) related to risk of osteoporosis in adulthood?	Longitudinal cohort study
3	Are the effects of early-life antibiotic exposure (birth to adolescence) on the gut microbiota related to PBM accrual and/or bone mineral density in later life?	Longitudinal cohort study
4	What are the effects of childhood illness on the development of a healthy gut microbiota and how does this affect PBM attainment and risk of osteoporosis in adulthood?	Case control studies, longitudinal cohort study
5	Can the negative impact of inflammatory diseases in childhood such as IBD, asthma, JIA on adult bone health be attenuated by manipulating the gut microbiota through diet, and pro- or prebiotic use?	Targeted exploratory and intervention studies in patient populations
6	Will recent changes in dietary habits such as vegan diets and gluten free diets in non coeliac individuals in younger generations affect future BMD and does this operate through alteration of the gut microbiota?	Case–control studies, Longitudinal cohort study
7	Can BMD loss be attenuated by the use of targeted dietary modification or supplementation with pre- or probiotics during the peri-menopausal time-span?	Clinical trials
8	Can a patient’s gut microbiota influence the individualized response to medications such as bisphosphonates, PTH, calcium and vitamin D supplements?	Prospective observational study (1–5 year duration)
9	Is the increased osteoporosis risk evident in underweight individuals mediated by the gut microbiota?	Longitudinal cohort study
10	How does the gut microbiota affect the availability and absorption of calcium, vitamin D and other mineral nutrients such as magnesium from the gut lumen?	Experimental animal and human studies
11	Can a specific or complex dietary modification such as an increased fibre, protein intake, Mediterranean or DASH diet, prebiotic or probiotic-use improve bone mineral density and at what stage in the lifecourse is the greatest benefit seen?	Clinical trials with long-term follow-up
12	Can short chain fatty acid supplementation (directly or via increased non-starch polysaccharide intake) improve BMD and if so, at what stage of the life-cycle?	Clinical trials
13	Is the reduction in BMD evident in the frail and elderly directly related to the concurrent decrease in gut microbial diversity that occurs in later life?	Cross-sectional, case–control studies, longitudinal cohort study

*IBD* inflammatory bowel disease, *IBS* irritable bowel syndrome, *JIA* juvenile idiopathic arthritis, *BMD* bone mineral density, *PBM* peak bone mass, *DASH* dietary approaches to stopping hypertension

It is also known that, as for many other complex traits, the inter-individual variability in microbiota composition is partly under genetic control, and the first such genetic factors have now been identified by work of the MiBiogen consortium [65]. This is the first global collaboration of various microbiome research groups to establish more harmonized procedures to combine and analyse microbiome data from different sources. This genetic analysis will lead to a better understanding of which biological factors and pathways are controlling origin, maintenance and perturbations in the microbiome population. In addition, genetic factors have been identified by Genome-Wide Association Studies for many of the physiological variables associated with microbiota composition. This opens up the possibility to perform Mendelian Randomization studies to distinguish causal effects from associations arising due to confounding, and to inform intervention studies based on nutrition, lifestyle or therapeutics such as anti-, pro- or pre-biotics [66–68].

There are many unknowns about the effects of therapeutically administered microbes on bone. While it is important to investigate these effects, it is also salient to understand how factors such as nutrients (for example wholegrain foods and fibre), dietary patterns (for example vegetarian, vegan and mediterranean diets), medication and antibiotic use, as well as medical co-morbidities, may predispose to osteoporosis by exerting impact on the gut microbiome. Research in the microbiome field so far suggests that ‘making a good microbiome bad’ (for example by adopting a Westernized diet) is a lot easier than ‘making a bad microbiome good.’ This underlines the importance of advancing our knowledge of the impact of early life on development of the gut microbiome in relation to bone health.

## Design of Clinical Studies

The research priorities identified for progression of microbiome-osteoporosis research highlight a clear need for large, prospective observational studies with long-term follow-up. To expedite this research, utilization of existing cohorts may provide some of the required answers but in some cases microbiome data may not have been collected. Cohorts such as UK-BIOBANK and the Avon Longitudinal Study of Parents and Children (ALSPAC) in the United Kingdom represent large, well-established longitudinal databases that could be useful. For interventional clinical trials that manipulate the microbiota with the intent of host benefit, a follow-up time of less than 6 to 9 months is unlikely to generate useful data, such is the time it takes for persistent, meaningful changes to be enacted upon the adult microbiota. In studies aiming to prevent complications of osteoporosis such as fractures it remains unclear what the best suggorate measure should be as primary outcomes in clinical trials. Regardless of the chosen primary outcome, we suggest that such studies incorporate a minimum of 12-month participant follow-up as has been performed previously [19].

Many microbiome studies suffer from low statistical power, lack of replication and use of variable technologies to assess the microbiome. Studies with low sample sizes often represent pilot investigations, that under ideal circumstances may identify strong microbiome-phenotype correlations, but lack replication in independent settings or in larger cohorts. Interventional studies often recruit several hundred participants [11]. Very large microbiome studies, such as the American Gut Project (> 10,000 participants) [69], have the benefit of limiting technical variability as samples are usually treated uniformly, and the effects of confounding variables can be adjusted for in statistical models. Very large cohort studies may also facilitate the finding of ‘a needle in a hay-stack,’ increasing the probability of finding microbial effects that are not apparent amongst the *noise* of studies with smaller sample sizes [70]. Emphasis should be placed on replication of findings and this should be pursued through collaboration and replication cohorts, especially in the case of studies with smaller sample sizes.

An important consideration for all studies on the human gut microbiota, in particular association or interventional studies aimed at altering the characteristics of the microbiota, relates to the many confounding factors than can affect its composition and function. This highlights the importance of collecting data on key clinical variables that may directly affect the microbiota. These include recent antibiotic use, previous gastrointestinal surgery or illness, current medication use, smoking, alcohol intake, habitual intake of macro- and micro-nutrients, dietary supplementation, BMI and menopausal status. Adjustment for and consideration of these confounders underlines the importance of qualified biostatistical expertise and collection of sufficient and accurate metadata [71]. The availability of rich, linked metadata to microbiota sequences in data repositories is crucial to facilitate integrative, multi-study analysis across international groups and study population cohorts. We would encourage research groups to collect and submit detailed metadata and to make them available in a FAIR manner (Findable, Accessible, Interoperable, Re-usable).

## Technical Methodologies

Choice of technology to assess the microbiota composition and function is another critical consideration in microbiome research. Over the last 5 years, there has been a shift from compositional microbiome analysis using quantitative PCR and 16S rRNA sequencing. While 16S sequencing is especially useful for large-scale profiling of microbiome samples because of its low cost and robustness, the desire to increase our understanding of pathophysiology, has seen a move towards shotgun metagenomic sequencing that provides information on gene content and therefore functional “potential” of the microbiome. True functional insights can only be obtained by a combination of metagenomic analysis of the gut microbiome with other ‘omic technologies such as transcriptomics, proteomics or metabolomics, in combination with intervention studies and Mendelian Randomization studies.

While it may seem obvious, it is noteworthy to highlight the importance of standardized procedures at many levels in microbiome research. This begins with faecal sample collection and storage methods, as these processes can affect apparent microbiota composition and the ability to accurately use additional ‘omic technologies. Early consultation and collaboration with experienced biostatisticians and assuring availability of the appropriate software and bioinformatic pipelines are all essential components. For a more in-depth overview and further guidance, readers are directed to the referenced articles by Allaband et al. and Knight et al.[72, 73]

With respect to dietary assessment, the ideal and most accurate method for habitual dietary recording has long been debated (such as a 7-day food diary vs. semi-quantitative food frequency questionnaire). Like other recommendations in this section, the choice of dietary assessment tool depends on the hypothesis under investigation; the level of quantification required (frequency of food intake versus mass quantity of food); cost; time expended and use of the most accurate assessment tool within the context of the study design. Some research has suggested different dietary assessment methods capture distinct areas of diet-microbiome interaction, potentially arguing against a reliance on a single dietary tool [74]. The introduction of app- and web-based methods of dietary assessment, as well as emerging innovations in food image recognition and wearable technologies, promises to reduce participant burden and facilitate richer and more accurate dietary assessment. Depending on the research question at hand, it may be appropriate to consider methods for adjusting for total energy intake and energy under/over-reporting.

## Concluding Remarks

Understanding of how food and nutrition affect life processes is fundamental to improving health and preventing disease. With an increasing societal awareness of the gut microbiota and the continued emphasis on the impact of food and lifestyle on health, the complex interplay between nutrition, the gut microbiome and bone health is of interest to many. Methods of analysing the gut microbiome have advanced and are now well-positioned for us to investigate the mechanisms by which modern lifestyle, diet and illness exert their effects on bone health via the gut microbiome. Microbiome research offers exciting prospects for patients with osteoporosis. We believe the next decade will provide significant progress in this new field of osteo-microbiology.
